# Mental Fatigue in Patients with Hearing Loss and/or Tinnitus Undergoing Audiological Rehabilitation—A Pilot Study

**DOI:** 10.3390/jcm12216756

**Published:** 2023-10-25

**Authors:** Satu Turunen-Taheri, Per-Inge Carlsson, Elisabeth Ternevall, Sten Hellström

**Affiliations:** 1 Department of CLINTEC, Division of Ear, Nose and Throat Diseases, Karolinska Institutet, SE-141 86 Stockholm, Sweden; sten.hellstrom@ki.se; 2 Department of Audiology and Neurotology, Karolinska University Hospital, SE-118 95 Stockholm, Sweden; elisabeth.ternevall@gmail.com; 3 Department of CLINTEC, Division of Audiology, Karolinska Institutet, SE-141 52 Huddinge, Sweden; 4 Department of Otorhinolaryngology, Central Hospital, SE-652 30 Karlstad, Sweden; per-inge.carlsson@regionvarmland.se; 5 Faculty of Medicine and Health, Medicine and Health, Örebro University, SE-701 82 Örebro, Sweden

**Keywords:** tinnitus, comorbidities, extended audiological rehabilitation, hearing impairment, mental fatigue, mental fatigue scale (MFS), severity of tinnitus

## Abstract

Background: Both tinnitus and hearing loss are multidimensional. The purpose of this study was to identify and determine the degree of mental fatigue in patients with hearing loss and/or tinnitus participating in audiological rehabilitation, and to examine the self-reported mental fatigue scale (MFS) in this patient group. Methods: Patients undergoing audiological rehabilitation at the Department of Audiology and Neurotology, Karolinska University Hospital, Sweden, between 2011 and 2017 who completed a self-reported MFS questionnaire were investigated. Data on 76 patients were analysed in this pilot study. Patients were also assessed using the Tinnitus Handicap Inventory (THI). Results: The study population had an age range of 38–65 years, and most had normal hearing (37%) or mild to moderate hearing loss (46%). Only 17% had severe to profound hearing loss. A total of 56.5% had tinnitus, of whom 39.5% scored ≥57 on the THI, indicating severe tinnitus, whereas 43.5% reported no tinnitus. The MFS scores, ranging from 13 to 42.5 points, were divided into three severity levels for analysis: 10.5–15, 15.5–20, and ≥20.5. In total, 67% of the patients had MFS scores ≥ 20.5. Importantly, most of the participants (90%) with a THI score ≥ 57 belonged to that group. A significant positive correlation between a high MFS score and severe tinnitus was found. Conclusions: The study reveals that severe mental fatigue is more common in patients with severe tinnitus than sole hearing loss.

## 1. Introduction

Globally, hearing loss was the third leading cause of years lived with disability in 2019 and the second leading cause of impairment [1], affecting 1.5 billion people [2]. Communication difficulties related to hearing loss may lead to social isolation, reduced quality of life (QoL) [3,4], and increased depression symptoms [5].

Tinnitus is a troublesome condition that exerts negative effects on cognitive function, e.g., working memory and concentration [6]. Tinnitus may lead to a reduced health-related QoL affecting domains such as physical functioning, vitality, and mental health [7]. Most tinnitus studies have reported a prevalence of 10% to 15% in the adult population [8]. A systematic review [9] reported an overall prevalence between 5.1% and 42.7% and indicated that prevalence generally increased with age. About 90% of people with tinnitus also have hearing loss, though many may not even realize they have both conditions [10].

Fatigue or mental fatigue is a complex, subjective phenomenon to define. Fatigue is often described as “early exhaustion after physical or mental activity” related, e.g., to lack of energy, sleep deprivation, and fatigability [11]. Fatigue is common after chronic health conditions such as brain injury or neurological diseases [12]. Common symptoms of mental fatigue are problems with cognitive functioning such as concentration and memory, stress intolerance, light and noise sensitivity or intolerance, and reduced sleep. A patient with mental fatigue can become mentally exhausted, which is characterized by an abnormally long recovery time after cognitive activity [13]. Long-standing auditory processing impairment is associated with changes in brain activation and can cause mental fatigue over time [14]. Clinically, it is known that patients with hearing impairment become tired and exhausted due to the mental effort involved in perceiving speech and communicating [15]. Patients with hearing loss reported low energy/vigor and severe fatigue, but interestingly, this increased risk did not correspond to the degree of hearing loss [16]. Background noise also negatively affects the ability of patients with hearing loss to understand speech, which leads to communication difficulties [17] and may lead to mental fatigue [15]. A study by Jahncke and Halin [18] showed that patients with hearing impairment had higher levels of stress hormones and were more fatigued by noise exposure than patients with normal hearing. Tinnitus is often associated with hearing loss, but when it comes to the connection between tinnitus and mental fatigue, this relationship is not well studied. In a systematic review of 20 studies, Holman et al. [19] showed a lack of substantial evidence to support that hearing loss increases fatigue or that hearing aids decrease fatigue.

The validated self-reported mental fatigue scale (MFS) developed by Johansson et al. in 2010 [12] and adapted from Rödholm et al. in 2001 [20] (Supplementary Materials, MFS S1 and S2) has been used to evaluate the degree of long-lasting mental fatigue after brain injury or stroke. The MFS questionnaire consists of 15 multidimensional questions and addresses mental disorders (affective), cognitive and neurological (sensory) symptoms, and sleep. The questions cover fatigue in general, lack of initiative, mental fatigue, mental recovery, concentration difficulties, memory problems, slowness of thinking, sensitivity to stress, increased tendency to become emotional, irritability, sensitivity to light and noise, reduced or excessive sleep, and 24 h variations with better or worse problems (morning, afternoon, evening, or night) [12]. In patients with brain damage, the MFS provides a detailed and accurate description of the patient complaints and an opportunity to monitor the clinical course.

Patients with severe hearing loss and/or severe tinnitus should participate in an extended audiological rehabilitation program involving interdisciplinary rehabilitation with at least three hearing care professionals (an audiologist, physician, hearing rehabilitation educator, welfare officer, psychologist, or technician) and/or participate in group rehabilitation to experience the maximum benefits of the treatments available [21]. In Sweden, patients need a referral to participate in an extended audiological rehabilitation program at Karolinska University Hospital. Patients in the program have severe to profound hearing loss, severe vision impairment, severe tinnitus with or without hearing loss, sudden unilateral hearing loss/deafness, or an additional disability that affects communication, e.g., Meniere’s disease. Normal/basal audiological rehabilitation includes hearing aid fitting and psychosocial competence assessment by an audiologist as a comparison to extended audiological rehabilitation.

Since 2011, the self-reported MFS questionnaire has been used during the audiological rehabilitation of patients with hearing loss and/or patients with severe tinnitus at Karolinska University Hospital, Stockholm, Sweden. The present study is the first to report on the use of the MFS questionnaire in patients with hearing loss and tinnitus in Sweden.

The overall aim of the present pilot study was to use the MFS to identify and investigate the degree of mental fatigue caused by hearing loss and eventual tinnitus in patients undergoing audiological rehabilitation. Furthermore, the purpose was to examine the usefulness of a self-reported MFS as a diagnostic instrument for mental fatigue in these patient groups.

The main conclusions indicate a relationship between severe tinnitus and severe mental fatigue in patients with normal hearing or mild to moderate hearing loss. The study found a relationship between tinnitus and severe mental fatigue with consequences that affect negatively patients’ daily lives. It is important to create guidelines for audiological interventions and treatments for patients with mental fatigue in combination with hearing loss and tinnitus.

## 2. Materials and Methods

### 2.1. Study Population

Patients participating in extended audiological rehabilitation from 2011 to 2017 at the Department of Audiology and Neurotology, Karolinska University Hospital in Sweden who completed the self-reported MFS were included in the study. A mental fatigue score of 10.5 or more was the inclusion criterion. A score of 10.5 or more [22] in the self-reported MFS questionnaire is suggested [23] for the identification of symptomatic mental fatigue; see more details below. The exclusion criterion was a cut-off MFS of 10 or less based on no symptoms of mental fatigue [23]. The present pilot study had no control group.

**Figure 1:**
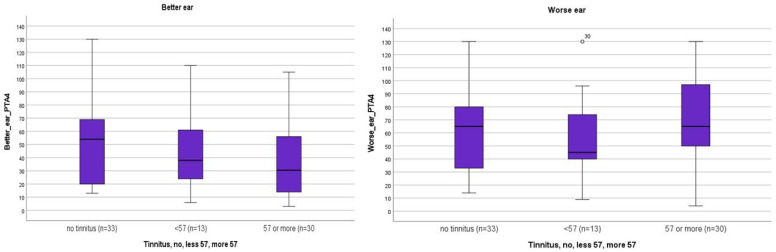
**Distribution of pure-tone air conduction hearing thresholds.** Distribution of the medians and percentiles of pure-tone air conduction hearing thresholds, PTAs (=PTA4) for a total of 76 patients in the better and the worse ear with Tinnitus Handicap Inventory (THI) score of no tinnitus (n = 33), <57 (n = 13), and ≥57 (n = 30) for a total of 76 patients. Better/worse ear median: no tinnitus (54/65), THI<57 (38/45), and ≥57 (31/65). Cronbach’s alpha 0.752 for internal reliability.

The present pilot study is based on data from 76 consecutive patient medical records. Of these, 23 (30.5%) were men and 53 (69.5%) were women. Information on the study and a consent form were sent to all 76 patients, all of whom signed consent forms. Table 1 shows the demographic characteristics of the 76 patients who had an MFS score ≥ 10.5 stratified by gender. The mean pure-tone air conduction hearing averages (PTA4) at frequencies of 0.5 Hz, 1 kHz, 2 kHz, and 4 kHz with hearing level (HL) in decibels (dB) for the 76 patients are shown in Figure 1.

The known diagnoses for hearing impairment in the study group varied, such as hearing impairment in childhood diagnosed in 15 (19.5%) patients, genetic causes in 3 (4%), Ménière’s disease in 4 (5.5%), otosclerosis in 10 (13%), chronic otitis in 7 (9%), meningitis in 3 (4%), rubella in 2 (2.5%), and ototoxic causes in 2 (2.5%). There were other diagnoses such as in two cases a radical mastoidectomy, two patients with vestibular schwannoma, two with labyrinthitis, three with sudden deafness, and in three cases facial nerve paralysis. Some of the patients had other problems in addition to hearing loss or tinnitus, such as 9% having previous head trauma, whiplash injury, or other problems with the neck or back; 15.5% having psychiatric symptoms or diagnosed depression; 5% having dizziness or balance difficulties; and 4% having fibromyalgia.

Several patients suffered from severe tinnitus when they visited our clinic (Table 1), which sparked our interest in studying the Swedish version of the MFS in a population of patients with hearing loss and tinnitus.

### 2.2. A Self-Assessment Questionnaire for Mental Fatigue—MFS

The validated MFS contains 15 questions evaluating affective, cognitive, and sensory symptoms. Each question is responded to by selecting one of four levels (0–3): no (0), mild (1), moderate (2), and severe (3) problems (Supplemental Materials in both MFS S1 Swedish and MFS S2 English version). A cut-off score of 10.5 is suggested for the sum of the results of questions 1 to 14 [23], with a total score of 0–10 indicating no symptoms, 10.5–15 indicating mild/slight symptoms, 15.5–20 indicating moderate/fairly serious symptoms, and 20.5 or more indicating severe/serious symptoms [24]. Question 15 pertains to the changes in symptoms over the last 24 h.

### 2.3. Tinnitus Handicap Inventory (THI)

The self-reported THI score [25] was used to define patients with tinnitus in this study. The THI contains 25 questions about the consequences of tinnitus and hearing difficulties related to tinnitus and is used to quantify the impact of tinnitus on daily life. Patients could answer yes (4), sometimes (2), or no (0), and the total score was categorized as follows: slight or no (0–16), mild (18–36), moderate (38–56), severe (58–76), or catastrophic (78–100) handicap. A classification indicating severe handicap was based on a cut-off score of 57 points or more according to the Stockholm region’s consultation requirements founded according to the guidelines for severe tinnitus [26].

### 2.4. Statistical Analysis

Statistical analyses were performed using IBM^®^ SPSS^®^ Statistics version 27 (IBM, Armonk, NY, USA). The value of the MFS questionnaire as a diagnostic instrument was evaluated. The MFS data, hearing data from medical records, sick leave information, data on the various types of audiological rehabilitation received, and factors such as sex and age were analysed. Continuous data were analysed using unpaired *t*-tests, and categorical data were analysed using chi-square tests. The means, medians, standard deviations, and percentages were calculated. The study divides the MFS scores into three levels based on questions 1–14: 10.5–15, 15.5–20, and ≥20.5. The data do not have to be normally allocated in linear regression if the number of subjects is above 30 [27]. Survey responses are not quota data, but linear regression suggests that such analysis is reasonable.

The study used statistical analyses such as correlation analysis and the Kruskal–Wallis test to better understand the interactions between variables. Next, a principal component analysis (PCA) was performed with the MFS score as the dependent variable to identify the relationship between the MFS and THI score. Pearson’s correlation coefficient (*R*) analysis was performed on the MFS score, PTA, and THI. A on-parametric Kruskal–Wallis test was performed to check the differences between various independent groups (THI < 57, THI ≥ 57 and no tinnitus) and a dependent MFS score.

## 3. Results

### 3.1. Demographic Data of All Included Subjects Stratified by Gender

The demographic data of the 76 patients in the study population, stratified by gender, are shown in Table 1. There were 23 (30.5%) males and 53 (69.5%) females, with an age range of 38–65 years and a mean age of 52.6 (SD 6.06) years. Most patients (48.5%) were in the 51 to 60 year old age group, of whom 11 (29.7%) were men and 26 (70.3%) were women. The second largest age group was the 41 to 50 year olds, accounting for 33% of the patients, of whom 9 (36%) were men and 16 (64%) were women. A total of 23.5% (*n* = 18) of the patients, 6 men (33.3%) and 12 women (66.7%), lived alone.

Most of the participants (*n* = 28, 37%) had PTAs of normal hearing or moderate hearing loss at 41–70 dB HL (*n* = 22, 29%) in the better ear. There were 13 patients (17%) with severe to profound hearing loss (71–90 dB HL and 91–130 dB HL), and another 13 patients (17%) had mild hearing loss.

There were 43 participants (56.5%) that reported problems with tinnitus, of whom 30 patients (39.5%), 8 (35%) men and 22 (41.5%) women, had severe tinnitus, based on a THI score of 57 or more (Table 1). There were 33 patients (43.5%) that reported no tinnitus at all. Only seven patients with a THI score ≥ 57 had normal hearing in both ears.

There were three sub-groups in the study population: patients with normal hearing or mild to moderate hearing loss without amplification; patients with hearing loss and hearing aids; and patients with hearing loss and cochlear implants (CI). See Section 3.2. for more information.

### 3.2. Hearing Aids and Cochlear Implants (CIs)

Of the total 76 patients, 57 (75%) had bilateral hearing aids, and 2 (2.6%) had bilateral CIs. A total of seven (9.2%) patients had a unilateral hearing aid, and four (5.3%) had hearing aid in combination with a CI on the opposite ear. Finally, six (7.9%) patients did not have any kind of aid at all. These six patients had tested hearing aids or stimulators for tinnitus but returned them due to no use of them.

### 3.3. Demographic Data Stratified by MFS Level: 10.5–15, 15.5–20, and ≥20.5

The mean MFS score was 23.4 (SD 5.7). The scores ranged from 13 to 42.5 points, with a median of 23.0 points. Most patients (*n* = 51, 67%) had an MFS score of 20.5 or more. In total, 11 (14.5%) scores were ≥30, and all of them had a high THI score of 78–100 points. The distribution of the 76 patients across the three MFS scores (10.5 to 15; 15.5 to 20; and ≥20.5) is shown in Table 2. The proportions of patients with scores between 10.5 to 15 and 15.5 to 20 were 6.5% and 26.5%, respectively.

In the group with the most severe fatigue symptoms, MFS scores ≥ 20.5, the age ranged from 38 to 65 years, which was almost the same as the ranges in the other score intervals, although the MFS score interval 10.5–15 included some elderly individuals.

There were 17 patients (33.5%) with normal hearing (0–25 dB HL) and MFS scores greater than 20.5, 9 patients (45%) had MFS scores between 15.5 and 20, and only 2 patients (40%) had MFS scores between 10.5 and 15. A similar distribution was observed in the patients with moderate hearing loss (41–70 dB HL). The six patients (8%) with profound hearing loss (91–130 dB HL) were distributed equally between the MFS 15.5–20 and ≥20.5 groups.

The distributions of occupation and sick leave across the three MFS score groups are shown in Table 2. Most of the participants (*n* = 68, 89.5%) were working, although 44 (58%) of them were on sick leave when they arrived at the clinic. During ongoing rehabilitation at the clinic, 73 of them (96%) were on sick leave. According to the medical records, a total of 30% of the study population worked in a school or preschool, 29% worked in administration or IT, and 12% worked in healthcare.

The severity of tinnitus, indicated by a THI ≥ 57, THI < 57, or no tinnitus, was also investigated across the three MFS score groups (Table 2). Of the patients with THI ≥ 57 (*n* = 27), 53% were in the MFS score ≥ 20.5 group; the difference was significant (*p*-value 0.002). However, seven patients (13.5%) in the THI < 57 group were also in the MFS score ≥ 20 group. Patients with no tinnitus but with hearing impairment mostly had MFS scores greater than 20.5 (*n* = 17, 33.5%).

### 3.4. Hearing Thresholds for the Entire Study Group in the Better and Worse Ear, with or without Tinnitus

The mean hearing thresholds with pure-tone air conduction averages (PTAs) at four frequencies (500, 1000, 2000, and 4000 Hertz = PTA4) were 44.1 dB HL in the better ear (SD 29.23) (median: 38 dB HL. Range: 3–130 dB HL) and 64.8 dB HL in the worse ear (median: 64 dB Hl. Range: 4–130 dB HL).

Figure 1 shows the distribution of the averages in the PTA4 (medians and percentiles) in the better and worse ear in patients with or without tinnitus. The results indicate a tendency in the better ear for patients with severe tinnitus, ≥57, to have a lower median (31) PTA4 than patients with less tinnitus, <57 (38), or no tinnitus at all (54). In the worse ear, the median PTA (65) for severe tinnitus and no tinnitus at all was the same, and for patients with a THI score less than 57, the median PTA4 was 45. No statistical analysis was performed for the distribution.

### 3.5. Correlation Analysis on Variables MFS, PTA4, and THI

**Figure 2:**
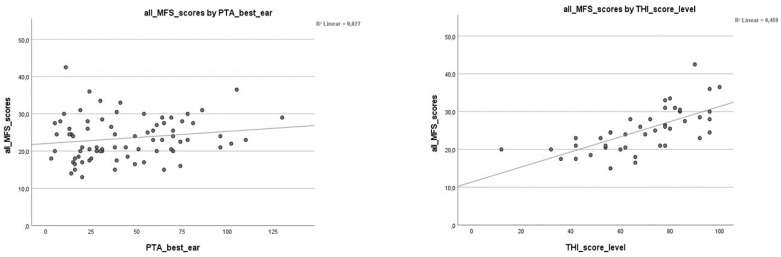
**Correlation of MFS scores, PTA4 and THI.** Correlation, r2 linear, between mental fatigue scale (MFS) scores and PTA4, and tinnitus score levels. All MFS scores by PTA4 better ear r = 0.027, all MFS scores by THI score level r=0.459.

The correlation analysis in Table 3 confirmed the relationship between all MFS scores and tinnitus with a significant positive correlation *r* = 0.677 **. The MFS score levels were separated (10.5–15, 15.5–20, and ≥20.5) and correlated significantly for moderate MFS (15.5–20) and severe MFS (≥20.5) scores with the PTA4 for the better ear and tinnitus scores. No significant correlation was detected for mild MFS (10.5–15). Figure 2 demonstrated a correlation between the MFS score and the PTA4 for the better ear, explained in Table 3, amounting to only 2.7% (*r* = 0.027) of the total variance in the MFS score. For the THI scores, a mild *r* = 0.45 explained about 45.9% of the total variance in MFS score.

To check the differences between various independent groups (THI < 57, THI ≥ 57, and no tinnitus) and dependent MFS scores, a Kruskal–Wallis test was performed. The Kruskal–Wallis test confirms the relationship between MFS scores and a THI score ≥ 57, corresponding to an increasing trend in the MFS score when tinnitus increases with a positive correlation *ρ* = 0.002 ** (Table 4). Furthermore, a decreasing relationship appears between MFS score and no tinnitus with *ρ* = 0.030 *, corresponding to the MFS score reducing when the patient is not bothered by tinnitus (Table 4).

**Figure 3:**
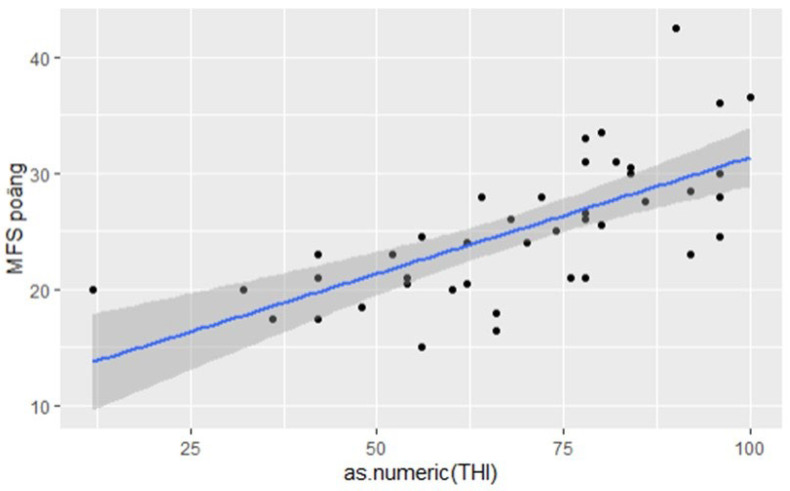
**The linear regression analysis of THI and MFS.** The linear regression analysis with least squares relationship between THI and MFS scores in patients where THI measurement was indicated upon referral. The slope was 0.2 MFS points per THI score with an intercept at 11.3 MFS points. The relationship was significant (*p* < 0.0001) with a correlation (Pearson’s R2) of 0.45.

The relationship between the THI and MFS scores is confirmed in the linear regression analysis with the slope of 0.2 MFS points per THI score with an intercept at 11.3 MFS points. The relationship was significant (*p* < 0.0001) with a correlation (Pearson’s R^2^) of 0.45 (Figure 3).

**Figure 4:**
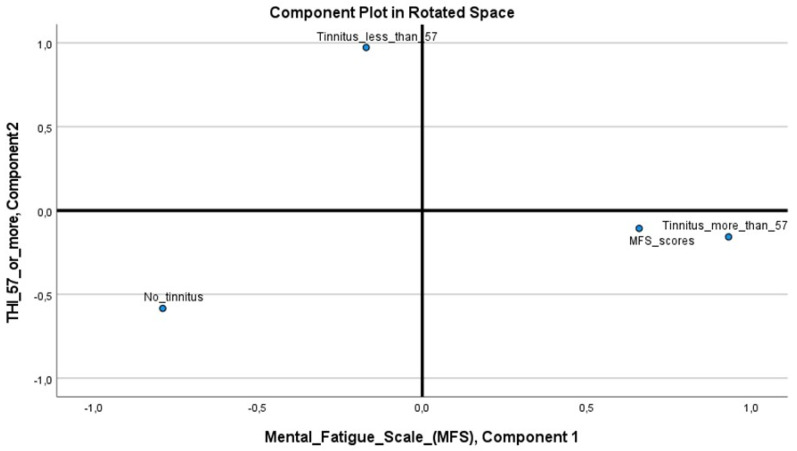
**PCA of MFS and THI.** Principal component analysis (PCA) with mental fatigue scale scores (MFS) and relationship with THI ≥ 57, THI < 57, and no tinnitus at all. Pattern matrix (Rotation Method: Oblimin with Kaiser normalization) showed MFS scores: 0.660, and THI ≥ 57: 0.932, no tinnitus: —0.584, and THI < 57: 0.973. The total variance were explained with 33% with THI ≥ 57, and MFS scores with 49%.

Principal component analysis (PCA) (Figure 4) confirmed the relationship between the MFS score and a THI score ≥ 57. The first component, the MFS score, explained 49% of the variation. The second component, a THI score ≥ 57 factor, explained 33% of the variation.

## 4. Discussion

To our knowledge, this is one of the first attempts to use the Swedish version of the MFS self-assessment questionnaire to assess patients with hearing loss and/or tinnitus. Severe mental fatigue was found among patients with normal hearing, as well as with mild to moderate hearing loss. Interestingly, a strong relationship between severe MFS scores and tinnitus were confirmed. Patients with severe tinnitus (≥57) seemed to have better hearing thresholds (PTAs) than patients with less tinnitus (<57) or no tinnitus in their better ear, as shown in Figure 1.

Three sub-groups were identified: patients without amplification, patients with hearing aids, and patients with CIs. The amplification type was dependent on whether patients had normal hearing with tinnitus, mild/moderate hearing loss, or severe hearing loss. The mental fatigue reported by patients in each of these groups could have been influenced by various sources, for example, their hearing situation, their amplification type or lack thereof, or the impact of tinnitus, if present.

Notably, our findings indicated that 51 of 76 patients (67%) had a severe MFS score of 20.5 or more. Strikingly, most patients with the highest MFS scores had severe tinnitus. Apparently, severe tinnitus is associated with serious effects on mental fatigue. It is also noteworthy that of these, 11 patients had MFS scores above 30. All 11 patients had severe tinnitus with THI scores between 78–100, and only 2 of them had normal hearing. Similar to the present study, an earlier study showed no relationship between the degree of hearing loss and fatigue, but it did not study tinnitus [16].

To date, the MFS instrument has not been validated for use in assessing mental fatigue in patients with hearing loss with or without tinnitus. A recent publication by the team at Vanderbilt outlines the development and validation of a new measure of listening-related fatigue [28]. A recent systematic review of 20 studies on hearing loss and fatigue, most using a Likert scale or dichotomous questions (yes/no), found that the majority of the included studies did not find significant results to support the hypothesis that hearing loss and hearing aid fitting has an impact on fatigue; even a meta-analysis was not possible due to the heterogeneity and number of studies [19]. Various scales are available, but there is no consensus on a “gold-standard” measure of subjective fatigue, particularly in patients with audiologic difficulties. The results in the present study differ from the results of the systematic review, and one can speculate on whether the contradictory findings may be due to the difference in study populations, ours including patients with severe tinnitus, or due to the choice of questionnaire, ours being the MFS.

Hornsby et al. [29] concluded that “the mechanisms responsible for hearing loss-related fatigue, and the efficacy of audiologic interventions for reducing fatigue, remain unclear”. Information suggesting a possible relationship between fatigue and hearing loss and/or tinnitus is subjective and is mainly derived directly from anecdotal reports or indirectly from related qualitative research [30,31,32]. A recent study [33] demonstrated a model of negative effects from high levels of listening-related efforts in adults with hearing loss, including physical, emotional, cognitive, and social areas of fatigue, e.g., exhaustion, lack of energy, and tiredness.

Mental fatigue is commonly associated with brain injuries. The MFS scale used in the present study was developed and validated by Johansson et al. [12] for use in patients with these disorders. An example of the usefulness of MFS in Sweden is its capacity to assess mental fatigue status related to sick leave. Mental fatigue affects a patient’s ability to work, leading patients to need to take sick leave, sometimes for long periods, to rest away from work and the attendant sound. The MFS was shown to provide valuable information during the follow-up of these patients that could be communicated to the Swedish Social Insurance Agency (Försäkringskassan, FK).

The study population in the present pilot underwent extended audiological rehabilitation and consisted of 76 patients with varying degrees of mental fatigue. Most patients in the present study were between 41 and 60 years of age at the time they were referred to receive hearing healthcare at the clinic. Most of the patients included in the study with mental fatigue were women (69.5%). One can speculate on the tendency for gender differences in mental fatigue. Women may be more willing to seek help for their audiological disturbances than men. An earlier study found women received extended audiological rehabilitation significantly more often compared to men [21]. It was also shown that MFS scores increased with increasing age. Almost all patients (89.5%) worked: 6 out of 10 in school as teachers or after-school teachers, 3 in administration or IT, and 1 of 10 in healthcare. Obviously, certain occupations seem to predispose individuals to the mental fatigue caused by hearing loss and/or tinnitus. This observation needs further research.

Taking individual questions into account regarding mental fatigue/fatigability, mental recovery, concentration, slowness of thinking, stress sensitivity, sensitivity to noise, and reduced sleep, severe tinnitus appears to have serious consequences on patients’ daily lives. A previous study pointed out that severe tinnitus and vertigo strongly and negatively affected QoL [34]. Similarly, the present study showed that a combination of tinnitus and/or hearing loss with mental fatigue negatively affects daily life. Patients tended to score high for stress sensitivity, which was assessed with question 8 on the MFS, indicating that patients with a high MFS score were intolerant to stress.

Some limitations to the study could be addressed. First, the present pilot study is based on retrospective data previously collected during routine clinical appointments. Second, it was not possible to sort the patients into separate groups representing either tinnitus or hearing loss because many patients had both tinnitus and hearing loss. Only seven patients had normal hearing, but they had a THI score ≥ 57, while some of the patients had no hearing loss in the better ear, but they had hearing loss in the worse ear. An attempt to differentiate between these groups was one of the purposes of the final analysis of the overall patient dataset in terms of the MFS scores. This could not be realized in the present study. Third, patients were not separated in the analysis based on whether they had amplification (hearing aids or CI) or not. This was because only two patients had bilateral CIs and four patients had a CI in combination with a hearing aid. A follow-up study is planned with a larger sample and this limitation can then be mitigated. Fourth, one of the aims of the study was to determine the usefulness of the MFS as a diagnostic instrument. A potential limitation of questionnaires such as the MFS is that it is based on self-reporting and cannot distinguish between the impact of severe tinnitus and severe anxiety, which might manifest similarly in terms of mental fatigue. Finally, this pilot study did not have a control group. The study did not include patients with low mental fatigue scores (<10.5), or people in general with hearing loss and tinnitus, and does not utilize any other self-reporting measure previously used in hearing loss populations as a comparison. The authors plan to include a wider population, including a control group, in a follow-up study.

Applying knowledge from earlier studies using the MFS instrument, a score of 10.5 or more implicates problems with mental fatigue that are likely to affect a patient’s daily life, including work and/or social activities [23]. Mental fatigue is pronounced fatigue from which patients cannot rest, and a long time is needed for recovery, which means that it differs markedly from ordinary fatigue [23]. Patients with mental fatigue function at a lower mental level, are sensitive to stress, and have difficulty concentrating. Hence, it is necessary to evaluate and quantify fatigue to monitor their clinical progress. Strikingly, some of the patients in the present study presented with higher MFS values than those reported for patients with head injuries, compared to healthy controls [23]. In this context, one should also consider that a patient with hearing loss and/or tinnitus could be affected by another permanent or chronic conditions (comorbidity), which might explain the high MFS scores in this patient group. For example, in the present study, 15.5% had psychiatric symptoms or diagnosed depression; 5% had dizziness or balance difficulties; and 4% had fibromyalgia. Comorbidity is certainly an important factor affecting mental fatigue and therefore important to study in this context. However, the data in this study did not allow us to study comorbidities, and further studies with larger patient populations are needed in the future.

## 5. Conclusions

Most of the study population had serious problems with mental fatigue. Several had normal hearing, while others had mild, moderate, and severe to profound hearing loss. More than half of the study group had tinnitus. Of those with tinnitus, 4 of 10 had severe tinnitus, and 90% had MFS scores indicating that they had severe mental fatigue. The results of the present study indicate a strong relationship between mental fatigue and severe tinnitus.

Obviously, this is an area that has so far been often overlooked. The results of the present study indicate that the self-reported MFS seems to have multidimensional questions and could be used as a diagnostic instrument for this patient group, but further investigation is needed to find methods of hearing and/or tinnitus rehabilitation that can reduce or shorten the duration of mental fatigue. This is especially important in terms of the negative effect mental fatigue has on patients’ daily lives. The study indicates a benefit of the use of the MFS for both patients and clinics, but further studies are needed to validate the questionnaire.

Finally, our results lead to the conclusion that there is a need for specific guidelines on the treatment of patients with hearing loss and/or tinnitus who have mental fatigue.

## Figures and Tables

**Table 1:** Demographic data. Demographic characteristics in patients with mental fatigue score > 10.5 in Sweden with the proportions of male and female patients.

	Total	Male	Female
	*n* (%)	*n* (%)	*n* (%)
Gender; total	76 (100)	23 (30.5)	53 (69.5)
Age (years),	52.55	52.09	52.75
Mean (±SD)	(6.06)	(6.09)	(6.10)
Range (years)	38–65	43–65	38–63
Civil status, living alone ^a^	18 (23.5)	6 (33.3)	12 (66.7)
Age groups (years)			
19–30	0 (0)	0 (0)	0 (0)
31–40	3 (4)	0 (0)	3 (6)
41–50	25 (33)	9 (39)	16 (30)
51–60	37 (48.5)	11 (48)	26 (49)
61–70	11(14.5)	3 (13)	8 (15)
Degree of hearing loss, better ear			
0–25 dB HL ^b^	28 (37)	6 (26)	22 (41.5)
26–40 dB HL	13 (17)	3 (13)	10 (19)
41–70 dB HL	22 (29)	8 (35)	14 (26.5)
71–90 dB HL	7 (9)	4 (17.5)	3 (5.5)
91–130 dB HL	6 (8)	2 (8.5)	4 (7.5)
Tinnitus; THI ^c^ < 57	13 (17)	7 (30)	6 (11.5)
Tinnitus; THI ^c^ ≥ 57	30 (39.5)	8 (35)	22 (41.5)
No tinnitus	33 (43.5)	8 (35)	25 (47)
Extended audiological rehabilitation ^d^	68 (89.5)	22 (32.5)	46 (67.5)

^a^ No indication of civil status: 11 (14.5%) patients. ^b^ HL = Hearing level in decibels (dB); 0–25 dB HL = normal, 26–40 dB HL = mild, 41–70 dB HL = moderate, 71–90 dB HL = severe, and 91–130 dB HL = profound hearing loss. ^c^ THI = Tinnitus Handicap Inventory. Total of 43 (56.5%) patients with tinnitus (THI < 57 and THI ≥ 57). ^d^ Participated in group rehabilitation or rehabilitated at least with three various specialists.

**Table 2:** Demographic data stratified by MFS. Demographic characteristics in patients with mental fatigue stratified by three various MFS levels: 10.5–15, 15.5–20, and ≥20.5 in Sweden.

	Total	MFS Score	MFS Score	MFS Score
		10.5–15	15.5–20	≥20.5
	*n* (%)	*n* (%)	*n* (%)	*n* (%)
Gender; total	76	5 (6.5)	20 (26.5)	51 (67) ^a^
Female	53 (69.5)	4 (7.5)	13 (24.5)	36 (68)
Male	23 (30.5)	1 (4.5)	7 (30.5)	15 (65)
Age (years)				
Mean (±SD)	52.55 (6.06)	53.80 (4.76)	53.65 (6.18)	52.00 (6.15)
Range (years)	38–65	49–60	38–63	38–65
Civil status, living alone ^b^	18 (23.5)	2 (11)	4 (22)	12 (67)
Age groups				
(years)				
19–30	0 (0)	0 (0)	0 (0)	0 (0)
31–40	3 (4)	0 (0)	1 (5)	2 (4)
41–50	25 (33)	2 (40)	3 (15)	20 (39)
51–60	37 (48.5)	3 (60)	12 (60)	22 (43)
61–70	11 (14.5)	0 (0)	4 (20)	7 (14)
Degree of hearing loss, better ear ^c^				
0–25 dB HL	28 (37)	2 (40)	9 (45)	17 (33.5)
26–40 dB HL	13 (17)	1 (20)	1 (5)	11 (21.5)
41–70 dB HL	22 (29)	1 (20)	5 (25)	16 (31.5)
71–90 dB HL	7 (9)	1 (20)	2 (10)	4 (7.5)
91–130 dB HL	6 (8)	0 (0)	3 (15)	3 (6)
Occupation				
Unemployed	7 (9)	1 (20)	1 (5)	5 (10)
Working	68 (89.5)	4 (80)	19 (95)	45 (88)
Sick leave permanent	1 (1.5)	0 (0)	0 (0)	1 (2)
Sick leave ^d^	44 (58)	2 (4.5)	14 (32)	28 (63.5)
Sick leave ^e^	73 (96)	4 (5.5)	20 (27.5)	49 (67)
Tinnitus; THI ^f^ < 57	13 (17)	1 (20)	5 (25)	7 (13.5)
Tinnitus; THI ^f^ ≥ 57	30 (39.5)	0 (0)	3 (15)	27 (53)
No tinnitus	33 (43.5)	4 (80)	12 (60)	17 (33.5)
Extended audiological rehabilitation ^g^	68 (89.5)	4 (6)	17 (25)	47 (69)

^a^ 11 (14.5%) had MFS score ≥ 30. ^b^ No indication of civil status: 11 (14.5%) patients. ^c^ HL = Hearing level in decibels (dB); 0–25 dB HL = normal, 26–40 dB HL = mild, 41–70 dB HL = moderate, 71–90 dB HL = severe, and 91–130 dB HL = profound hearing loss. ^d^ On sick leave at the time of seeking healthcare at the clinic. ^e^ On sick leave at the time as a patient in the clinic. ^f^ THI = Tinnitus Handicap Inventory. ^g^ Participated in group rehabilitation or rehabilitated at least with three various specialists.

**Table 3:** Correlation analysis of MFS score, PTA4, and THI score. Correlation analysis for all mental fatigue scale (MFS) scores, and association between PTA4 levels for the better ear, THI scores, and also the separate MFS score levels (10.5–15, 15.5–20, 20.5 or more) associated with the PTA4 for the better ear and THI scores, with Pearson’s correlation coefficient (*r*) with 95% confidence intervals (CI).

	PTA4 ^a^ Better Ear	Tinnitus ^b^
		(THI)
	*n* = 76	*n* = 76
MFS all scores		
Pearson’s *R*	0.165	0.677 **
95% CI	(−0.091–0.389)	(0.564–0.790)
*p*-value	0.153	0.000 **
MFS *mild* (10.5–15)		
Pearson’s *R*	−0.155	−0.222
95% CI	(−0.296–0.009)	(−0.367—0.079)
*p*-value	0.182	0.054
MFS *moderate* (15.5–20)		
Pearson’s *R*	−0.231 *	−0.270 *
95% CI	(−0.396−0.032)	(−0.480—0.065)
*p*-value	0.045 *	0.018 *
MFS *severe* (20.5 or more)		
Pearson’s *R*	0.298 **	0.370 **
95% CI	(0.116–0.460)	(0.164–0.559)
*p*-value	0.009 **	0.001 **

** Correlation is significant at the 0.01 level (two-tailed). * Correlation is significant at the 0.05 level (two-tailed). ^a^ PTA4 means the average pure-tone thresholds, i.e., at frequencies of 0.5, 1, 2, and 4 kHz at various degrees of hearing loss in better ear: 0–25 dB HL, 26–40 dB HL, 41–70 dB HL, 71–90 dB HL, and 91–130 dB HL. ^b^ All tinnitus (THI) scores.

**Table 4:** The relationship between MFS scores and tinnitus. Analysis of all mental fatigue scale (MFS) scores and tinnitus in 76 patients. A non-parametric Kruskal–Wallis test was performed to check variance in differences between various independent groups (THI < 57, THI ≥ 57, and no tinnitus) and dependent MFS groups (10.5–15, 15.5–20, ≥20.5).

	THI ≥ 57 *n* = 30	THI < 57 *n* = 13	No Tinnitus *n* = 33
MFS scores ^a^			
10.5–15 mean rank (*n* = 5)	23.50	39.60	52.40
15.5–20 mean rank (*n* = 20)	29.20	41.50	44.80
20.5 > mean rank (*n* = 51)	43.62	37.22	34.67
*p*-value ^b^	0.002 **	0.521	0.030 *

^a^ Grouping variable: MFS_scores. ^b^ * *p* < 0.05; ** *p* < 0.01.

## Data Availability

Data available on request due to restrictions eg privacy or ethical, The data presented in this study are available on request from the corresponding author. The data are not publicly available due to ethical restictions.

## Supplementary Materials

The following supporting information can be downloaded at https://www.mdpi.com/article/10.3390/jcm12216756/s1, MFS S1: Swedish version of a self-assessment questionnaire for mental fatigue, MFS S2: English version of a self-assessment questionnaire for mental fatigue; is validated and contains 15 questions evaluating affective, cognitive, and sensory symptoms. Each question is responded to by selecting one of four levels (0–3): no (0), mild (1), moderate (2), and severe (3) problems.
