# Supplementary material for: Mental Fatigue in Patients with Hearing Loss and/or Tinnitus Undergoing Audiological Rehabilitation—A Pilot Study
Source: J Clin Med. 2023 Oct 25;12(21):6756. doi: 10.3390/jcm12216756 (PMC10648212; doi:10.3390/jcm12216756)
Supplement: Supplementary file 1 [file jcm-12-06756-s001.zip › jcm-2645441-supplementary.pdf]

Självskattning av mental trötthet/hjärntrötthet
Mental Fatigue Scale, MFS

Namn:_________________________ Datum: ______________

Vi är intresserade av ditt nuvarande tillstånd, d.v.s. ungefär hur du har mått **den senaste månaden**.
När du ska jämföra med **hur det var tidigare** ska du göra det med **hur det var innan du blev sjuk/skadades**.
I tabellen för varje fråga finns fyra påståenden som beskriver *Inga* (0), *Lätta* (1), *Medelsvåra* (2) och *Svåra* besvär (3).

Vi vill att du markerar den siffra som står bredvid det påstående som bäst beskriver dina besvär.
Om du tycker att du hamnar mellan två påståenden finns det även siffror som motsvarar detta.

1. Trötthet

Har du känt dig trött den senaste månaden? Det spelar ingen roll om det är fysisk (muskulär trötthet) eller trött i huvudet. Om det nyligen hänt något ovanligt (t.ex. en olycka eller tillfällig sjukdom) skall du försöka bortse från det.

0	Jag har inte alls känt mig trött (aldrig onormalt trött, inte behövt vila mer än vanligt).
0.5	
1	Jag har varit trött flera gånger per dag, men jag blir klart piggare av att vila.
1.5	
2	Jag har känt mig trött större delen av dagen, och vila har ingen eller liten effekt.
2.5	
3	Jag har känt mig trött all vaken tid, och vila har ingen effekt.

2. Oföretagsamhet

Har du svårt att sätta igång med saker? Känner du dig oföretagsam och tar det emot när du skall sätta igång med något, oavsett om det är en ny uppgift eller om det gäller saker du gör varje dag.

0	Jag har inga svårigheter med att ta itu med saker.
0.5	
1	Jag har svårare än tidigare för att sätta igång med aktiviteter. Jag skjuter gärna på det.
1.5	
2	Det krävs en stor ansträngning för att jag skall ta itu med saker. Detta gäller även vardagliga ting som att stiga ur sängen, tvätta mig, äta.
2.5	
3	Jag kan inte få de enklaste vardagliga saker (äta, klä på mig) gjorda. Jag måste ha hjälp med allt.

3. Mental Uttröttbarhet

Blir du snabbt trött "i huvudet" när hjärnan måste arbeta? Blir du mentalt trött av saker som att läsa, titta på TV eller delta i samtal med flera personer. Måste du ta pauser eller byta aktivitet?

0	Jag kan hålla på lika länge som vanligt. Min uthållighet för "hjärnarbete" har inte minskat.
0.5	
1	Jag blir lättare trött men kan utföra lika mycket "hjärnarbete" som är normalt för mig.
1.5	
2	Jag blir lätt trött och måste ta pauser eller göra något annat oftare än vanligt.
2.5	
3	Jag har så lätt för att bli trött att jag inte kan göra någonting, eller måste avbryta alla aktiviteter efter en kort stund (ca 5 minuter).

För mer information om MFS, se www.mf.gu.se eller www.brainfatigue.se

4. Mental återhämtning

Hur lång tid tar det för dig att återhämta dig efter att du har arbetat tills du fullständigt tappat förmågan att kunna koncentrera dig på det du gör.

0	Jag behöver mindre än en timmes vila för att kunna fortsätta arbeta.
0.5	
1	Jag måste vila mer än en timme, men behöver inte en natts sömn.
1.5	
2	Jag behöver en natts sömn för att kunna fortsätta arbeta.
2.5	
3	Jag behöver flera dagars vila för att återhämta mig.

5. Koncentrationssvårigheter

Har du svårt att samla tankarna och koncentrera dig?

0	Jag har lika lätt som vanligt för att samla tankarna.
0.5	
1	Jag kan ibland tappa bort mig, t.ex. när jag läser eller tittar på TV.
1.5	
2	Jag har så svårt att koncentrera mig så att det besvärar mig när jag t.ex. läser en dagstidning eller deltar i samtal med flera personer.
2.5	
3	Jag har alltid så svårt att koncentrera mig att det är nästan omöjligt att göra någonting.

6. Minnesstörningar

Glömmer du oftare än tidigare och behöver du minneslappar, eller måste leta mer hemma eller på arbetet?

0	Jag har inga problem med minnet.
0.5	
1	Jag glömmer saker lite oftare än vad jag tycker att jag borde, men kan klara mig om jag använder minneslappar.
1.5	
2	Mitt dåliga minne orsakar regelbundet besvär (t.ex. genom att jag glömmer viktiga möten eller spisen).
2.5	
3	Jag kan nästan inte komma ihåg någonting.

7. Tanketröghet

Känner du dig trög eller långsam i tankearbetet? Detta gäller känslan av att det tar ovanligt lång tid för att avsluta en tankegång eller för att lösa en uppgift som kräver tankearbete.

0	Jag känner mig inte trög eller långsam i mina tankar vid ”hjärnarbete”.
0.5	
1	Jag kan känna en viss tröghet någon eller några gånger om dagen vid krävande tankearbete.
1.5	
2	Jag känner mig ofta trög och långsam i tanken även vid vardagliga sysslor t.ex. i samtal med en person eller vid läsning av dagstidningen.
2.5	
3	Jag känner mig alltid väldigt trög och långsam i tanken.

8. Stresskänslighet

Har du haft svårt att hantera stress, alltså att göra många saker samtidigt och under tidspress?

**0**	Jag klarar stress lika bra som vanligt**.**
**0.5**	
**1**	Jag är mer lättstressad, men bara i krävande situationer som jag tidigare klarade av.
**1.5**	
**2**	Jag blir stressad lättare än vanligt. Det krävs mindre stressade situationer än tidigare för att jag skall känna av det.
**2.5**	
**3**	Jag har väldigt lätt för att bli stressad. Så fort som situationen är ovan eller påfrestande känner jag mig stressad.

9. Ökad känslosamhet

Har du ovanligt lätt för att gråta? Faller du lätt i gråt när du t.ex. ser en sorglig film eller när du pratar med dina anhöriga. Om det nyligen hänt något ovanligt (t.ex. en olycka eller sjukdom) skall du försöka bortse från det.

**0**	Jag är inte mera känslig än tidigare.
**0.5**	
**1**	Jag har en ökad känslighet som fortfarande är naturlig för mig. Jag har lätt att börja gråta eller får tårar i ögonen, men det händer bara för ting som engagerar mig starkt.
**1.5**	
**2**	Min känslighet är besvärande eller generande. Det händer att jag börjar gråta även för saker jag egentligen inte bryr mig om. Jag försöker undvika vissa situationer på grund av detta.
**2.5**	
**3**	Min känslighet orsakar stora problem för mig. Den stör min dagliga relation även i den nära familjen och gör att jag har svårt att klara mig utanför hemmet.

10. Irritabilitet eller "Kort stubin"

Är du ovanligt lättretad eller lättirriterad för saker som du tidigare tyckte var bagateller.

**0**	Jag är inte mer lättretad eller irritabel än tidigare.
**0.5**	
**1**	Jag blir lätt irriterad men det går fort över.
**1.5**	
**2**	Jag blir väldigt fort irriterad för bagateller eller för saker som andra inte bryr sig om.
**2.5**	
**3**	Jag reagerar med en intensiv ilska, eller känsla av raseri. Jag har svårt att behärska den.

11. Överkänslighet för ljus

Är du känslig för starkt ljus?

**0**	Jag har ingen ökad känslighet för ljus.
**0.5**	
**1**	Ibland kan jag ha svårt för starkt ljus som t.ex. solljus, reflexer från snö eller vatten eller glasrutor, starka lampor inomhus, men det kan lätt avhjälpas, t.ex. med solglasögon.
**1.5**	
**2**	Jag är så känslig för ljus att jag föredrar att uträtta mina dagliga aktiviteter i dämpad belysning. Jag har svårt att gå ut utan solglasögon.
**2.5**	
**3**	Min ljuskänslighet är så svår att jag inte kan gå ut utan solglasögon. Jag har ständigt neddragna gardiner (eller motsvarande).

För mer information om MFS, se www.mf.gu.se eller www.brainfatigue.se

12. Överkänslighet för ljud
Är du känslig för ljud?

**0**	Jag besväras inte av någon ökad känslighet för ljud.
**0.5**	
**1**	Ibland kan jag ha svårt för starka ljud (t.ex. musik, ljud från TV eller radion, eller plötsliga oväntade ljud), men det kan lätt åtgärdas genom att jag sänker ljudnivån. Min ljudkänslighet stör mig inte i mitt dagliga liv.
**1.5**	
**2**	Jag är klart ljudöverkänslig. Jag måste undvika starka ljud eller dämpa (t ex med öronproppar) dem för att klara mitt dagliga liv.
**2.5**	
**3**	Min ljudkänslighet är så svår att jag har svårt att klara mig hemma trots ljuddämpning.

13. Minskad nattsömn
Sover du dåligt om nätterna? Om din nattsömn har ökat, skattas detta som ”0”. Om du tar sömntabletter och sover normalt, skattas detta som 0.

**0**	Jag sover inte sämre än vanligt.
**0.5**	
**1**	Jag har lite svårt att somna eller min sömn är kortare, ytligare eller oroligare än normalt.
**1.5**	
**2**	Jag sover minst två timmar mindre än vanligt, och vaknar ofta på natten även utan yttre störningar.
**2.5**	
**3**	Jag sover mindre än två till tre timmar per natt.

14. Ökad sömn
Sover du mer och/eller djupare än vanligt? Om din sömn har minskat markeras detta som ”0”.
Obs! Räkna in även sömn dagtid.

**0**	Jag sover inte mer än vanligt.
**0.5**	
**1**	Jag sover längre eller tyngre, men inte så mycket som två timmar mer än vanligt, inklusive tupplurar.
**1.5**	
**2**	Jag sover längre eller tyngre. Minst två timmar längre än vanligt, inklusive tupplurar.
**2.5**	
**3**	Jag sover längre eller tyngre. Minst 4 timmar längre än vanligt och behöver dessutom sova dagtid.

Summera ihop fråga 1 till 14
En summa på 10,5 poäng eller över visar problem med hjärntrötthet och bör utredas vidare.

15. **Dygnsvariation.** Finns det tider på dygnet då de besvär vi frågat om (t ex trötthet, koncentration) är bättre eller sämre? Med regelbundet menar vi här åtminstone 3-4 dagar i veckan.

**0**	Jag har inte märkt att mina besvär är regelbundet bättre eller sämre vid vissa tider
**1**	Det finns en klar skillnad mellan olika tider på dygnet. Jag kan säga att jag kommer att må bättre vid en viss tid, och sämre vid andra tider.
**2**	Jag mår dåligt under all tid under hela dygnet.

Om det finns en dygnsvariation:

När mår du som *bäst?* Eftermiddagen/ Kvällen/ Natten
När mår du som *sämst*? Eftermiddagen/ Kvällen/ Natten

För mer information om MFS, se www.mf.gu.se eller www.brainfatigue.se

Mental Fatigue Scale, MFS

Name:_________________________ Date: ______________

We are interested in *your present condition*, that is, how you have felt during *the past month*.
When you are comparing your condition with "than before", compare it with how it was before the injury or getting ill.
Each question below is followed by four statements that describe: No (0), Slight (1), Fairly serious (2) and Serious (3) problems. We would like you to place a circle around the figure before the statement that best describes your problems. Should you find that your problem falls between two statements, there are also figures to indicate this.

1. Fatigue

Have you felt fatigued during the past month? It does not matter if the fatigue is physical (muscular) or mental. If you recently experienced something unusual (for example an accident or short illness) you should try to disregard it when assessing your fatigue.

0	I do not feel fatigued at all. (No abnormal fatigue, do not need to rest more than usual).
0.5	
1	I feel fatigued several times every day but I feel more alert after a rest.
1.5	
2	I feel fatigued for most of the day and taking a rest has little or no effect.
2.5	
3	I feel fatigued all the time and taking a rest makes no difference.

2. Lack of initiative

Do you find it difficult to start things? Do you experience resistance or a lack of initiative when you have to start something, no matter whether it is a new task or part of your everyday activities?

0	I have no difficulty starting things.
0.5	
1	I find it more difficult starting things than I used to. I'd rather do it some other time.
1.5	
2	It takes a great effort to start things. This applies to everyday activities such as getting out of bed, washing my self and eating.
2.5	
3	I can't do the simplest of everyday tasks (eating, getting dressed). I need help with everything.

3. Mental fatigue

Does your brain become fatigued quickly when you have to think hard? Do you become mentally fatigued from things such as reading, watching TV or taking part in a conversation with several people? Do you have to take breaks or change to another activity?

0	I can manage in the same way as usual. My ability for sustained mental effort is not reduced.
0.5	
1	I become fatigued quickly but am still able to make the same mental effort as before.
1.5	
2	I become fatigued quickly and have to take a break or do something else more often than before.
2.5	
3	I become fatigued so quickly that I can do nothing or have to abandon everything after a short period (approx. five minutes).

För mer information om MFS, se www.mf.gu.se eller www.brainfatigue.se

4. Mental recovery

How long do you need to recover after you have worked “until you drop” or are no longer able to concentrate on what you are doing?

0	I need to rest for less than an hour before continuing whatever I am doing.
0.5	
1	I need to rest for more than an hour but do not require a night’s sleep.
1.5	
2	I need a night’s sleep before I can continue whatever I am doing.
2.5	
3	I need several days rest in order to recover.

5. Concentration difficulties

Do you find it difficult to gather your thoughts and concentrate?

0	I can concentrate as usual.
0.5	
1	I sometimes lose concentration, for example when reading or watching TV.
1.5	
2	I find it so difficult to concentrate that I have problems, for example reading a newspaper or taking part in a conversation with a group of people.
2.5	
3	I always have such difficulty concentrating that it is almost impossible to do anything.

6. Memory problems

Do you forget things more often than before, do you need to make notes or do you have to search for things at home or at work?

0	I have no memory problems.
0.5	
1	I forget things slightly more often than I should, but I am able to manage by making notes.
1.5	
2	My poor memory causes frequent problems (for example forgetting important meetings or turning off the cooker).
2.5	
3	I can hardly remember anything at all.

7. Slowness of thinking

Do you feel slow or sluggish when you think about something? Do you feel that it takes an unusually long time to conclude a train of thought or solve a task that requires mental effort?

0	My thoughts are neither slow nor sluggish when it comes to work involving mental effort.
0.5	
1	My thoughts are a bit slow one or a few times each day when I have to do something that requires serious mental effort.
1.5	
2	My thoughts often feel slow and sluggish, even when carrying out everyday activities, for example a conversation with a person or when reading the newspaper.
2.5	
3	My thoughts always feel very slow and sluggish.

8. Sensitivity to stress

Do you find it difficult to cope with stress that is, doing several things at the same time while under time pressure?

**0**	I am able to cope with stress, in the same way as usual.
**0.5**	
**1**	I become more easily stressed, but only in demanding situations that I was previously able to manage.
**1.5**	
**2**	I become stressed more easily than before. I feel stressed in situations that previously did not bother me.
**2.5**	
**3**	I become stressed very easily. I feel stressed in unfamiliar or trying situations.

9. Increased tendency to become emotional

Do you find that you cry more easily than previously? Do you often burst into tears when, for example, you watch a sad film or talk with your family members? If you recently experienced something unusual (e.g. an accident or short illness) you should try to disregard it in your assessment.

**0**	I am not more emotional than I used to be.
**0.5**	
**1**	I am more emotional than other people but it is something that is natural for me. I start to cry or my eyes fill with tears easily, but only in relation to things that affect me deeply.
**1.5**	
**2**	My emotions are problematic or embarrassing. I sometimes even start to cry about things that mean nothing to me. I try to avoid certain situations because of this.
**2.5**	
**3**	My emotions cause me great problems. They disturb my day-to-day relationship with members of my immediate family and make it difficult for me to cope outside the home.

10. Irritability or "a short fuse"

Are you unusually short-tempered or irritable about things that previously did not bother you?

**0**	I am not more short-tempered or irritable than I used to be.
**0.5**	
**1**	I become more easily irritated, but it does not last very long.
**1.5**	
**2**	I become irritated very quickly about small things or things that do not bother other people.
**2.5**	
**3**	I react with extreme anger or rage, which I find very difficult to control.

11. Sensitivity to light

Are you sensitive to strong light?

**0**	I have no increased sensitivity to light.
**0.5**	
**1**	I sometimes experience problems with strong light such as sunlight reflected by snow, water or glass, or strong lights at home, but I am able to cope with it, for example by wearing sunglasses.
**1.5**	
**2**	I am so sensitive to light that I prefer to carry out my daily activities in dim light. I find it difficult to leave the house without sunglasses.
**2.5**	
**3**	My sensitivity to light is so strong that I am unable to leave the house without sunglasses. I keep the blinds (or equivalent) drawn at all times.

12. Sensitivity to noise
Are you sensitive to noise?

**0**	I do not suffer from increased sensitivity to noise.
**0.5**	
**1**	I sometimes have difficulty with loud noise (for example music, noise from the TV or radio or sudden, unexpected sounds), but I can deal with it easily by turning down the volume. My sensitivity to noise does not disturb my everyday life.
**1.5**	
**2**	I have a marked over-sensitivity to noise. I have to avoid loud noise or reduce it (for example by means of ear plugs) in order to cope with everyday life.
**2.5**	
**3**	My sensitivity to noise is so great that I find it difficult to manage at home despite sound insulation.

13. Decreased sleep at night
Do you sleep badly at night? If you are sleeping more than before at night, please place a circle around the ”0”. If you are taking sleeping tablets and sleep normally, please place a circle around ”0”.

**0**	I do not sleep less than before.
**0.5**	
**1**	I have slight problems falling asleep or my sleep is shorter, lighter or more restless than before.
**1.5**	
**2**	I sleep at least two hours less than before and wake up frequently during the night without anything disturbing me.
**2.5**	
**3**	I sleep less than two to three hours per night.

14. Increased sleep
Do you sleep longer and/or more deeply than before? If you are sleeping less than before, please place a circle around the ”0”. N.B. Please take account of time spent sleeping during the day.

**0**	I do not sleep more than usual.
**0.5**	
**1**	I sleep longer or deeper, but less than two hours more than usual, including naps during the day.
**1.5**	
**2**	I sleep longer or deeper. At least two hours more than usual, including naps.
**2.5**	
**3**	I sleep longer or deeper. At least four hours more than usual, and in addition I need to take a nap during the day.

Total score of item 1 to 14. A cutoff score at 10.5 is suggested. A score above 10 implicates problems with mental fatigue and the person should consider the actual situation with work or social life.

15. **24-hour variations (for clinical use).** Do you find that at certain times of the day or night the problems we asked about (for example tiredness, lack of concentration) are better or worse? In the statements below, “regularly” means at least 3 to 4 days of the week.

**0**	I have not noticed that my problems are regularly better or worse at certain times, or I do not have any specific problems.
**1**	There is a clear difference between certain times of the day. I can predict that I will feel better at certain times and worse at other times.
**2**	I feel unwell at all times of the day and night.

If you experience 24-hour variations:

When do you feel at your best?	Morning	Afternoon	Evening	Night
When do you feel at your worst?	Morning	Afternoon	Evening	Night

För mer information om MFS, se www.mf.gu.se eller www.brainfatigue.se
